# Protein-Protein Interactions Inferred from Domain-Domain Interactions in Genogroup II Genotype 4 Norovirus Sequences

**DOI:** 10.1155/2013/456356

**Published:** 2013-05-02

**Authors:** Chuan-Ching Huang, Chuan Yi Tang

**Affiliations:** ^1^Department of Computer Sciences, National Tsing Hua University, Hsinchu 30013, Taiwan; ^2^Department of Computer Sciences and Information Engineering, Providence University, Taichung 43301, Taiwan

## Abstract

Severe gastroenteritis and foodborne illness caused by Noroviruses (NoVs) during the winter are a worldwide phenomenon. Vulnerable populations including young children and elderly and immunocompromised people often require hospitalization and may die. However, no efficient vaccine for NoVs exists because of their variable genome sequences. This study investigates the infection processes in protein-protein interactions between hosts and NoVs. Protein-protein interactions were collected from related Pfam NoV domains. The related Pfam domains were accumulated incrementally from the protein domain interaction database. To examine the influence of domain intimacy, the 7 NoV domains were grouped by depth. The number of domain-domain interactions increased exponentially as the depth increased. Many protein-protein interactions were relevant; therefore, cloud techniques were used to analyze data because of their computational capacity. The infection relationship between hosts and NoVs should be used in clinical applications and drug design.

## 1. Introduction

Gastroenteritis and foodborne illness epidemics usually occur during the winter. In 1968, an acute gastroenteritis outbreak occurred at an elementary school in Norwalk, OH, USA [1]. Kapikian et al. used immune electron microscopy and an infectious stool filtrate to observe the virus particles and identified them as small round-structured viruses [2]. The nonbacterial pathogen was called the Norwalk virus after the outbreak region and renamed the Norovirus (NoV) at the International Congress of Virology in Paris in 2002. The NoV is the species of the genus of Norwalk-like viruses in the Caliciviridae family. 

The NoV consists of a positive-sense, single-strand RNA (ssRNA) genome sequence of approximately 7.5 kb with 3 open reading frames (ORFs) [3]. The first open reading frame (ORF1) contains approximately 5 kb of sequences, which may enable 195 kDa polyproteins to support virus duplication. During the duplication phase, polyproteins are divided into at least 6 nonstructural proteins, such as p48, nucleotide triphosphatase (NTPase), p22, Vpg, proteinase, and RNA-dependent RNA polymerase [4–6]. ORF2 is approximately 1.8 kb long, which may transform into 60 kDa major structural protein VP1 with the following functions: self-assembly and capsid formation, receptor recognition, host specificity, strain diversity, and immunogenicity [7]. ORF3 consists of 0.6 kb nucleotides, which may translate into a 20 kDa minor structural protein (VP2), which facilitates the expression and stability of major structural protein VP1 [4, 8].

The NoV structure is formed by 180 capsid protein monomers organized into 90 dimeric capsomers [9]. Each capsid protein consists of an N-terminal shell (S) domain and a C-terminal protrusion (P) domain. The S domain forms a contiguous 8-stranded beta-barrel shell of the capsid and is commonly found in the capsid proteins of other viral families. The arch-like structure extending from the shell is formed by the P domain, which can be composed of P1 and P2 subdomains. The P2 subdomain is the critical segment because of its location on the capsid surface and because it exhibits the most variable sequence. 

Because traditional cell cultures cannot replicate NoVs [10], the phylogenetic relationship is mainly investigated by using genome-sequencing techniques. Based on the phylogenetic analysis of genome sequences, 5 genogroups exist: GI, GII, GIII, GIV, and GV. GIII is found in cattle [11, 12], GV is found in mice [13], and human NoVs are divided into 2 major genogroups, GI and GII. 

High-throughput sequencing techniques allow several genome sequences to be investigated simultaneously. This enables the mutation tendency of diverse NoV sequences to be examined. The sequence search tool on the Pfam website was used to collect approximately 30 NoV GII genotype 4 sequences to find their corresponding Pfam domains. The 7 main families are Cacli_PP_N (PF08405), RNA helicase (PF00910), Peptidase_C37 (PF05416), RdRP_1 (PF00680), Calici_coat (PF00915), Calici_coat_C (PF08435), and RNA_capsid (PF03035). Because the P2 subdomain is associated with antigenicity by binding to histoblood group antigen receptors, the infection relationship may be hidden by the interactions of Pfam domains extracted from NoV sequences. This study attempts to distinguish the protein-protein interactions (PPIs) from the domain-domain interactions (DDIs) and to use the PPIs to determine the biological regulation of NoVs. Many PPIs are expected, requiring high-capacity computation.

Cloud computing enables high-capacity computation. Cloud computing allows data to be collaboratively computed by different computer systems. Three main types of cloud computing services exist: infrastructure as a service (IaaS), software as a service (SaaS), and platform as a service (PaaS). User requirements determine the type of service used. When managing many PPIs [14], the distributed characteristic of cloud techniques enhances the efficiency of exploring the biological regulation caused by NoVs. This provides an opportunity to examine the relationship between hosts and NoVs and to investigate clinical applications and drug design.

## 2. Materials and Methods

**Figure 1:**
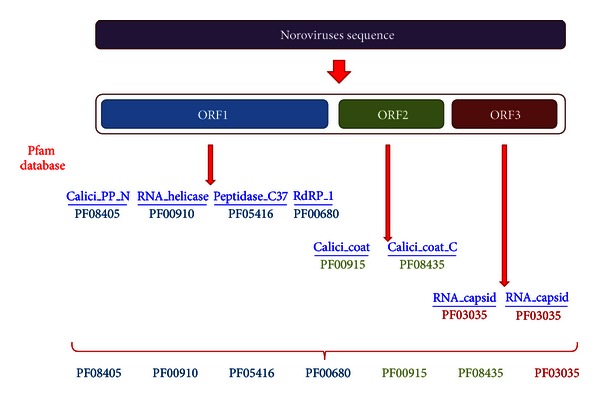
The domain composition of a NoV sequence which is aligned by the sequence search tool on the Pfam website.

Seven Pfam [15] domains were extracted from approximately 30 genome sequences from GII genotype 4 using the sequence search tool on the Pfam website. Four domains (PF08405, PF00910, PF05416, and PF00680) exist in ORF1 (Figure 1). Two domains (PF00915 and PF08435) exist in ORF2, and one domain (PF03035) exists in ORF3. 

**Figure 2:**
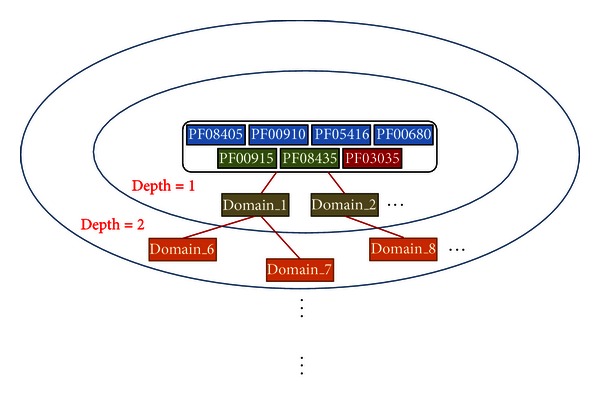
Illustration of DDIs from 7 NoV-related domains.

The DOMINE database (version 2.0) [16] was used to investigate interactions between domains, including known and predicted protein domain interactions. For each of the 7 NoV domains, if a related DDI was found in DOMINE, the interaction and interactive domains were recorded. The interactive domains and the 7 NoV domains were grouped into a domain set of Depth 1. The interactive domain set with interactions in the Depth 1 domain set was denoted as the domain set of Depth 2. This method was used to collect domain sets of increasingly higher depth (Figure 2). 

**Figure 3:**
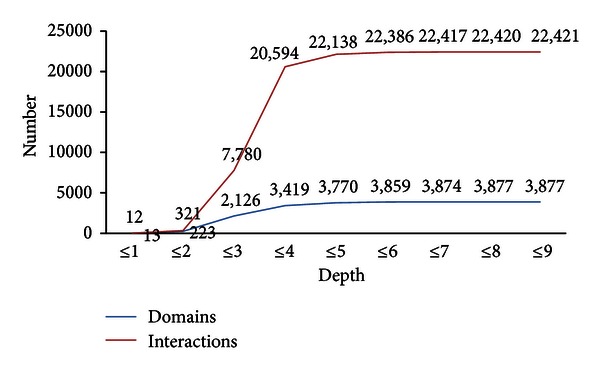
Population of domains and interactions within each depth.

The number of domains and DDIs increases as the depth increases. The domains and interactions from Depths 1 to 9 are shown in Figure 3. Depth 2 has 17 times more domains and 27 times more interactions than Depth 1. Depth 3 has 10 times more domains and 24 times more interactions than Depth 2. The number of domains and the number of interactions plateau gradually after Depth 4. 

**Figure 4:**
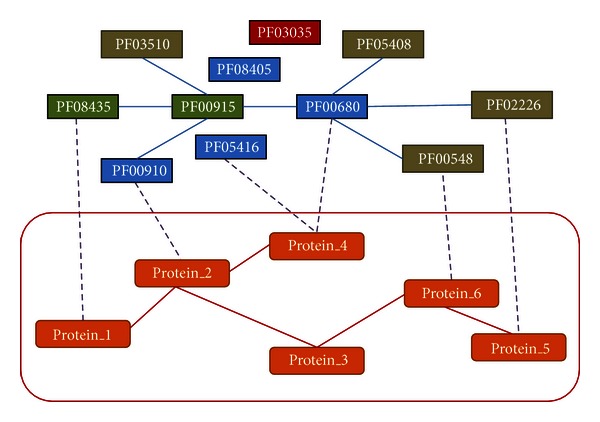
Illustration of the PPI network extending from DDIs within Depth 1.

The set of DDIs within each depth was used to locate corresponding proteins for each domain and to construct the putative PPI network.  Figure 4 shows the PPIs inferred from the DDIs within Depth 1. Corresponding proteins were collected by either using the NoV-related domains or by extending the domains within Depth 1 to build a PPI network to identify similar metabolic pathways. 

For each domain from a certain depth, all related proteins were extracted from the UniProt Knowledgebase (release 2011_07) [17]—a comprehensive database containing protein sequence and function information. Proteins were grouped by the Pfam domain (version 25.0) from the integrated source database, InterPro (version 33.0) [18]. 

An attempt was made to find connections between proteins based on the DDIs and to explore their accompanying protein functions. However, the protein interaction network was too complex to identify the proteins relevant to the NoVs. It would be beneficial to retain the most common PPIs that have been verified by the expert curators of the molecular interaction database, MINT [19]. Human proteins consisting of Pfam domains were selected to investigate the interaction relationship because the NoV uses people as hosts. 

## 3. Results

To examine the DDI strength, the depth is defined as the distance of a domain from the previous domain. Depth 1 includes 13 domains and 12 interactions (Figure 3). However, the number of domains associated with interactions increases by almost 17 times in Depth 2. The number of domains and interactions only increase slightly in Depth 4, meaning that the number of DDIs reached a boundary value.

**Figure 5:**
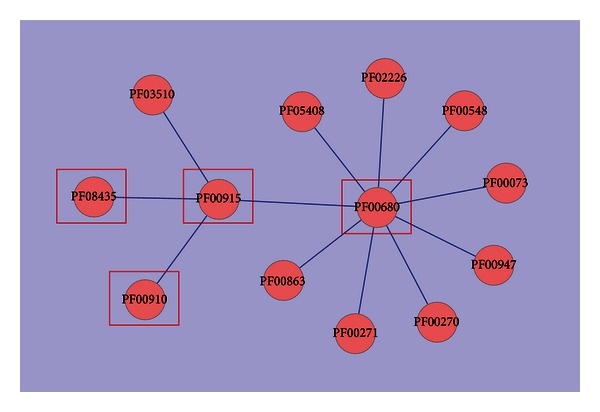
The domain-domain interaction network within Depth 1.

The DDIs in Depth 1 are illustrated using Cytoscape software [20] in Figure 5. Four Pfam domains (PF00910, PF00680, PF08435, and PF00915) were found in the interaction network of the 7 NoV-related domains. The first 2 domains are from ORF1, and the last 2 domains are from ORF2. 

**Figure 6:**
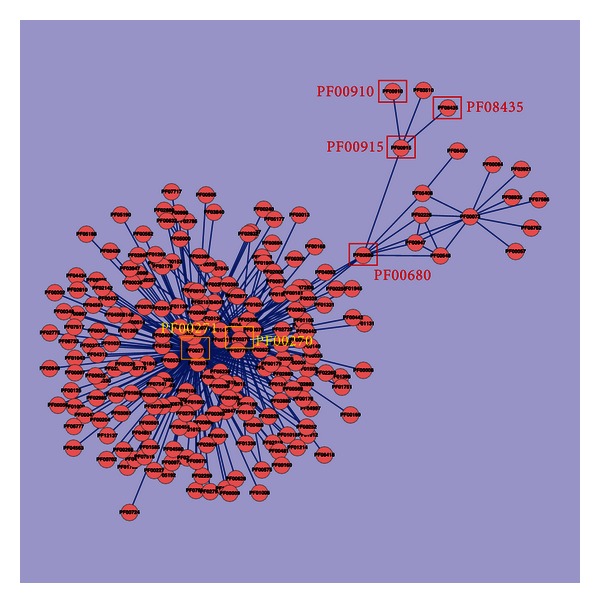
The domain-domain interaction network within Depth 2.

The DDIs in Depth 2 are more complex than those from Depth 1. The 4 NoV-related domains are shown in the top-right corner of Figure 6. The complex interactions are caused by 2 undirected domains (PF00270 and PF00271), which are centers for undirected domains and connect several related domains. 

To show the complexity of the PPI network from each depth, PPI statistics and distinct proteins are listed in Table 1. From Depth 2 to Depth 3, the number of PPIs increases rapidly, and the number of proteins almost triples. Of the 1061 proteins in Depth 2, 107 proteins have higher links into other proteins, including ATP-dependent RNA helicase, DNA annealing helicase and endonuclease, putative DNA repair and recombination protein, and the transcription termination factor. 

**Figure 7:**
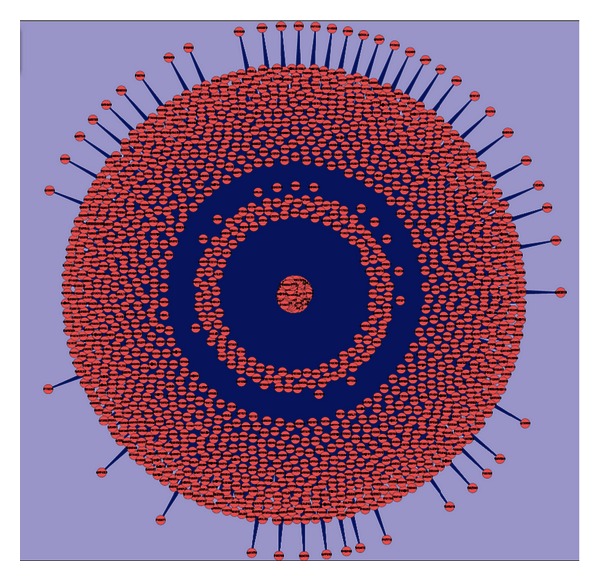
The protein-protein interaction network within Depth 2.

The PPI network from Depth 2 is shown in Figure 7 to show the interaction complexity between proteins. The 3 circles in the figure do not represent the proteins directly related to the 7 NoV domains and proteins in Depths 1 and 2. It is possible that proteins closer to the center have more putative linkages with other proteins. Infection may occur between NoVs and human hosts through some of these PPIs. 

## 4. Discussion

High-throughput genome-sequencing technologies generate vast amounts of sequence data efficiently and economically. Personalized medication could be produced by analyzing individual genome sequences. Domains are the fundamental building blocks of proteins [21]. They play important roles in living organisms such as catalysis, signal transduction, and the transport of nutrients [22]. This study uses the Pfam domain database. The database contains protein domain families that share significant degrees of sequence similarity. This approach assumes that one type of domain combination represents a protein and that the interaction between proteins could be performed through DDIs. Exploring protein regulation by using DDIs is reasonable.

Although domains are smaller units than proteins, extending PPIs from the domain level is feasible. This causes 2 main problems: the vast number of protein connections from DDIs and the ability to examine available PPIs for NoVs. Data integration and relationship establishment require extremely complex computations. Cloud computing techniques are available to overcome these obstacles. Applying biological knowledge to organism specificity and infection pathways would assist in comprehending NoVs. This may help researchers develop medical therapies for NoVs. 

## 5. Conclusions

Chemical reactions in living systems are performed by proteins. Understanding protein regulation may assist with understanding disease progression. Domains are basic protein folding and evolution units; therefore, investigating internal DDI relationships is valuable. For this study, we extracted NoV-related domains from the Pfam database to investigate the biological processes during NoV infection phases.

NoVs can cause acute gastroenteritis and foodborne illnesses, affecting lives and increasing health care costs. However, no efficient vaccine exists for NoVs. Exploring the regulation of PPIs extending from domains may help identify the relationship between hosts and NoVs. This information could be used in clinical applications and drug design. 

## Figures and Tables

**Table 1:** The statistics of PPIs and proteins within each depth.

Depth	Number of PPIs	Number of proteins
2	185,321	1,061
3	1,347,151	3,774
4	1,483,271	4,420
5	1,486,662	4,735
6	1,486,679	4,736
7	1,488,835	5,172
8	1,488,954	5,291
9	1,492,222	5,291

## References

[1] Adler JL, Zickl R (1969). Winter vomiting disease. *Journal of Infectious Diseases*.

[2] Kapikian AZ, Wyatt RG, Dolin R, Thornhill TS, Kalica AR, Chanock RM (1972). Visualization by immune electron microscopy of a 27-nm particle associated with acute infectious nonbacterial gastroenteritis. *Journal of Virology*.

[3] Jiang X, Wang J, Graham DY, Estes MK (1992). Detection of Norwalk virus in stool by polymerase chain reaction. *Journal of Clinical Microbiology*.

[4] Scipioni A, Mauroy A, Vinjé J, Thiry E (2008). Animal noroviruses. *Veterinary Journal*.

[5] Belliot G, Sosnovtsev SV, Mitra T, Hammer C, Garfield M, Green KY (2003). In vitro proteolytic processing of the MD145 Norovirus ORF1 nonstructural polyprotein yields stable precursors and products similar to those detected in calicivirus-infected cells. *Journal of Virology*.

[6] Hardy ME (2005). Norovirus protein structure and function. *FEMS Microbiology Letters*.

[7] Chen R, Neill JD, Noel JS (2004). Inter- and intragenus structural variations in caliciviruses and their functional implications. *Journal of Virology*.

[8] Bertolotti-Ciarlet A, Crawford SE, Hutson AM, Estes MK (2003). The 3' end of norwalk virus mRNA contains determinants that regulate the expression and stability of the viral capsid Protein VP1: a novel function for the VP2 protein. *Journal of Virology*.

[9] Prasad BVV, Hardy ME, Dokland T, Bella J, Rossmann MG, Estes MK (1999). X-ray crystallographic structure of the Norwalk virus capsid. *Science*.

[10] Duizer E, Schwab KJ, Neill FH, Atmar RL, Koopmans MPG, Estes MK (2004). Laboratory efforts to cultivate noroviruses. *Journal of General Virology*.

[11] Ando T, Noel JS, Fankhauser RL (2000). Genetic classification of ’Norwalk-like viruses’. *Journal of Infectious Diseases*.

[12] Koopmans M, Von Bonsdorff CH, Vinjé J, De Medici D, Monroe S (2002). Foodborne viruses. *FEMS Microbiology Reviews*.

[13] Karst SM, Wobus CE, Lay M, Davidson J, Virgin HW (2003). STAT1-dependent innate immunity to a norwalk-like virus. *Science*.

[14] Suravajhala P, Sundararajan VS (2012). A classification scoring schema to validate protein interactors. *Bioinformation*.

[15] Sonnhammer ELL, Eddy SR, Durbin R (1997). Pfam: a comprehensive database of protein domain families based on seed alignments. *Proteins-Structure Function and Genetics*.

[16] Yellaboina S, Tasneem A, Zaykin DV, Raghavachari B, Jothi R (2011). DOMINE: A comprehensive collection of known and predicted domain-domain interactions. *Nucleic Acids Research*.

[17] Magrane M, Consortium U (2011). UniProt Knowledgebase: a hub of integrated protein data. *The Journal of Biological Databases and Curation*.

[18] Apweiler R, Attwood TK, Bairoch A (2001). The InterPro database, an integrated documentation resource for protein families, domains and functional sites. *Nucleic Acids Research*.

[19] Ceol A, Chatr Aryamontri A, Licata L (2009). MINT, the molecular interaction database: 2009 update. *Nucleic Acids Research*.

[20] Smoot ME, Ono K, Ruscheinski J, Wang PL, Ideker T (2011). Cytoscape 2.8: new features for data integration and network visualization. *Bioinformatics*.

[21] Chothia C, Gough J, Vogel C, Teichmann SA (2003). Evolution of the protein repertoire. *Science*.

[22] Rost B, Liu J, Nair R, Wrzeszczynski KO, Ofran Y (2003). Automatic prediction of protein function. *Cellular and Molecular Life Sciences*.

